# Editorial Note: Cdc7 is a potent anti-cancer target in pancreatic cancer due to abrogation of the DNA origin activation checkpoint

**DOI:** 10.18632/oncotarget.28902

**Published:** 2026-08-28

**Authors:** Matthew T. Huggett, Slavica Tudzarova, Ian Proctor, Marco Loddo, Margaret G. Keane, Kai Stoeber, Gareth H. Williams, Stephen P. Pereira

**Affiliations:** ^1^UCL Institute for Liver and Digestive Health and UCL Cancer Institute, University College London, London, UK; ^2^The Research Department of Pathology, UCL Cancer Institute, University College London, London, UK; ^3^Oncologica Ltd, The Science Village, Chesterford Research Park, Cambridge, UK

The Oncotarget Scientific Integrity Office checked a data misplacement in the article: Huggett MT, Tudzarova S, Proctor I, Loddo M, Keane MG, Stoeber K, Williams GH, Pereira SP. Cdc7 is a potent anti-cancer target in pancreatic cancer due to abrogation of the DNA origin activation checkpoint. Oncotarget. 2016 Apr 5;7(14):18495-507. doi: 10.18632/oncotarget.7611. PMID: 26921250. PMCID: PMC4951304.

Specifically:

Figure 5D: The Control DAPI image for the TUNEL assay (IMR-90//CO non-targeting siRNA) overlaps with the Control DAPI image (IMR-90//CO treated with DMSO) in Figure 6B.

Figure 6B: A higher magnification section of the Control DAPI image for Capan-1 cells treated with DMSO in Figure 6B was used as the Control DAPI image for Panc-1 cells in Figure 6B, and appears again as the Capan-1/CO non-targeting siRNA image in Supplementary Figure 3D.

The Scientific Integrity Office has completed its analysis and found no additional inconsistencies. We have concluded that these image misplacements do not affect the article’s conclusions. The DAPI images serve as a control for the TUNEL assay, and the occurrence of apoptosis in the analyzed cell lines was also confirmed by flow cytometry analysis and western blotting for apoptosis-related proteins activation (Figures 3, 5 and 6).

We were unable to reach the authors of the article. Because the identified issues cannot be corrected with original data but do not affect the article’s conclusions, the journal is issuing an Editorial Note. This action is being taken in accordance with COPE guidance* to inform readers of the image irregularities and to maintain the transparency and integrity of the journal’s records.

*COPE guidance:“Concerns raised by a complainant that do not warrant a correction or expression of concern could also be resolved by some other means (e.g., letter to the editor, or note or notification).” https://publicationethics.org/guidance/guideline/expressions-concern.

Original article: Oncotarget. 2016; 7:18495–18507. Cdc7 is a potent anti-cancer target in pancreatic cancer due to abrogation of the DNA origin activation checkpoint. https://doi.org/10.18632/oncotarget.7611

